# Occupational Health: Shift Work–Cancer Debate Goes On

**DOI:** 10.1289/ehp.115-a535a

**Published:** 2007-11

**Authors:** M. Nathaniel Mead

Working the night shift disrupts the normal circadian rhythms of the body. This work pattern has been linked in some studies with cardiovascular disease, metabolic syndrome, and various cancers. Other findings to date have been either conflicting or of borderline significance. The debate goes on, with a study published online 7 September 2007 ahead of print in the *Scandinavian Journal of Work, Environment and Health* finding no evidence for an association between shift work and risk for any cancer, with the possible exception of thyroid cancer.

The study included about 3.2 million Swedish citizens who worked at least part-time. About 4.0% of the men and 0.4% of the women reported being shift workers, meaning they worked a rotating schedule or their schedule included working between 1:00 and 4:00 a.m. Occupations were categorized depending on the percentage of people engaged in shift work.

The researchers classified shift workers as people working in an industry with at least 40% shift workers (the exposed group). They compared the cancer incidence among this group to that of groups in which fewer than 30% declared themselves shift workers. The final results for these two groups showed no relationship between shift work and an increased risk of developing cancer. When the analyses were restricted to 70% of workers who said they worked rotating or night shifts, there was a 35% increase in the incidence of thyroid cancer for men only (the sample size for women was too small to achieve statistical significance).

The coauthors acknowledge their study had serious limitations, beginning with the fact that individual jobs were aggregated according to the degree of shift work reported by workers in each occupational category. “Because exposure was measured by the percentage of shift workers in a particular occupation rather than by individual shift worker, the relationship of cancer to individual exposures could not be directly determined but had to be inferred from the exposure of the occupational group,” says lead author Judith Schwartzbaum, an associate professor of epidemiology at The Ohio State University.

Some circadian scientists are adamant that shift work history should be based on individual exposures, not on aggregate measures according to occupation category. “Because of the likelihood of substantial amount of misclassification of the exposure of interest [i.e., night shift work], these results are not compelling evidence of the absence of an association,” says epidemiologist Scott Davis of the Fred Hutchinson Cancer Research Center. However, the study found no evidence for an effect of shift work even when the study’s classification of shift workers included only 30% of nonshift workers and the comparison group was restricted to occupations with 100% nonshift workers.

Cancer epidemiologist Richard Stevens of the University of Connecticut Health Center adds that the study’s null findings seem particularly dubious given consistent positive findings from numerous studies that were based on what he terms much better exposure assessment exposure data. Yet coauthor Maria Feychting, an epidemiologist based at the Karolinska Institute in Stockholm, Sweden, counters, “There are large differences between [earlier] studies in how exposure was defined, particularly how long duration of exposure is needed before an increased risk is evident; some studies find an increased risk already after a few years of exposure, whereas other studies require twenty to thirty years of shift work before any risk increase is evident.”

Several of the studies with positive findings are also based on aggregate measures of exposure, and although misclassification of the exposure is likely a problem in such studies, Feychting says, publishing only those with positive findings would give a false impression of consistency. She adds, “Whether there is an association between shift work and cancer is to me still an open question and further studies are needed.”

The study was also unable to control or adjust for known risk factors for cancer except for marital status and socioeconomic status. “Such factors represent potential confounders in the present study and may help explain why the current findings are inconsistent with the majority of published studies, a half-dozen of which have suggested an increase in breast cancer risk among female shift workers,” says Davis. Schwartzbaum concurs but also counters that results in previous studies did not change materially after adjustment for known risk factors.

“What’s needed at this point is a large cohort study including women from various occupations and prospectively collected individual information on working hours as well as potential confounding factors,” says Feychting. She suggests that future studies should also include detailed analyses of working hours, not just whether employees work multiple shifts, because shift work does not necessarily involve working hours during the night.

